# Foundational Nutrition: Implications for Human Health

**DOI:** 10.3390/nu15132837

**Published:** 2023-06-22

**Authors:** Jeremy R. Townsend, Trevor O. Kirby, Tess M. Marshall, David D. Church, Adam R. Jajtner, Ralph Esposito

**Affiliations:** 1 Research, Nutrition, and Innovation, Athletic Greens International, Carson City, NV 89701, USA; trevor.kirby@athleticgreens.com (T.O.K.); tess.marshall@athleticgreens.com (T.M.M.); ralph.esposito@athleticgreens.com (R.E.); 2 Department of Kinesiology, Lipscomb University, Nashville, TN 37204, USA; 3 Department of Geriatrics, Center for Translational Research in Aging & Longevity, Donald W. Reynolds Institute on Aging, University of Arkansas for Medical Sciences, Little Rock, AR 72205, USA; dchurch@uams.edu; 4 Exercise Science and Exercise Physiology, Kent State University, Kent, OH 44240, USA; ajajtner@kent.edu; 5 Department of Nutrition, Food Studies, and Public Health, New York University-Steinhardt, New York, NY 10003, USA

**Keywords:** foundational nutrition, human longevity, gut microbiome, diet, synergy

## Abstract

Human nutrition, and what can be considered “ideal” nutrition, is a complex, multi-faceted topic which many researchers and practitioners deliberate. While some attest that basic human nutrition is relatively understood, it is undeniable that a global nutritional problem persists. Many countries struggle with malnutrition or caloric deficits, while others encounter difficulties with caloric overconsumption and micronutrient deficiencies. A multitude of factors contribute to this global problem. Limitations to the current scope of the recommended daily allowances (RDAs) and dietary reference intakes (DRIs), changes in soil quality, and reductions in nutrient density are just a few of these factors. In this article, we propose a new, working approach towards human nutrition designated “Foundational Nutrition”. This nutritional lens combines a whole food approach in conjunction with micronutrients and other nutrients critical for optimal human health with special consideration given to the human gut microbiome and overall gut health. Together, this a synergistic approach which addresses vital components in nutrition that enhances the bioavailability of nutrients and to potentiate a bioactive effect.

## 1. Introduction: The Nutritional Health Problem

Poor nutrition is a pervasive problem worldwide, impacting people of all backgrounds and ages. According to the World Health Organization (WHO), nearly one in three people globally suffers from some form of malnutrition, whether it is undernutrition, micronutrient deficiencies, or overweight and obesity [1]. Furthermore, these nutritional inadequacies have a profound effect on human health and vitality beyond simple nutrient deficiency diseases. As early as 1981, it was estimated that approximately 30–35% of cancer deaths in the USA were linked to diet in an article written stemming from a commission from the United States (US) congress [2]. Over the past 40 years, this problem persists as one in five deaths worldwide, primarily from cardiovascular disease and cancer, can be attributed to a suboptimal diet [3]. While the link between nutritional habits and physical health has been widely acknowledged, striking evidence suggests that our dietary choices have a profound effect on our mental well-being [4,5]. Large observational studies have detected associations between dietary habits, mental health, and well-being while numerous nutrition and supplementation interventions have yielded beneficial effects on indices of depression, anxiety, and quality of life [6,7,8,9,10,11].

Given the severity of this nutritional health crisis, a multi-faceted approach is required, including policy changes to improve food environments and sustainable agricultural practices, increased access to healthy foods, and nutrition education to promote healthy dietary habits. This review will explore the scientific underpinnings of Foundational Nutrition and propose it as a new model by which researchers and practitioners approach and implement nutritional interventions. Therefore, the purpose of this review is three-fold: (1) propose a new working definition of “Foundational Nutrition”, a term we will propose as a means for individuals to achieve their daily essential nutritional needs while setting the stage for human health and performance (e.g., cognitive, digestive, musculoskeletal, cardiovascular, etc.); (2) outline the specific areas in which the conventional approach to nutrition may need reconsideration and can be addressed by a new paradigm; (3) explore nutritional concepts and selected nutrients which may be essential for humans to achieve optimal nutritional health and improve their healthspan. Undeniably, issues related to socioeconomic status (e.g., food insecurities, restricted access to healthy foods, limited monetary funds) and regional climate (e.g., declining agricultural productivity, food availability and seasonality, alterations in nutrient quality of crops, global climate change) largely contribute to the potential inability to meet the nutritional needs of an individual which are covered in-depth elsewhere [12,13,14,15,16]. This discussion is not intended to be all-encompassing; instead, it serves as a starting point for scientific discourse and further exploration of nutrition to empower human vitality to thrive instead of simply providing the rudimentary needs for survival.

## 2. Foundational Nutrition Definition

The human body is a complex biological system with countless biochemical and physiologic reactions occurring every second. For a system so complex, the traditional approach to nutritional science has been to identify and manipulate these processes by isolating the system of interest, providing a targeted intervention, and subsequently evaluating the outcome—essentially this is the reductionistic scientific method we can attribute numerous medical and health advancements we have today [17]. However, due to the ever-increasing health crisis which does not appear to be improving, the nutritional sciences need to re-evaluate the way nutrients and nutrient requirements are assessed within our intricate biological systems. One can argue that we are attempting to fit the nutritional sciences into the reductionistic scientific model, rather than attempting to assess a new method to identify how a complex system can be matched by a very comprehensive nutritional approach [17,18]. 

**Figure 1:**
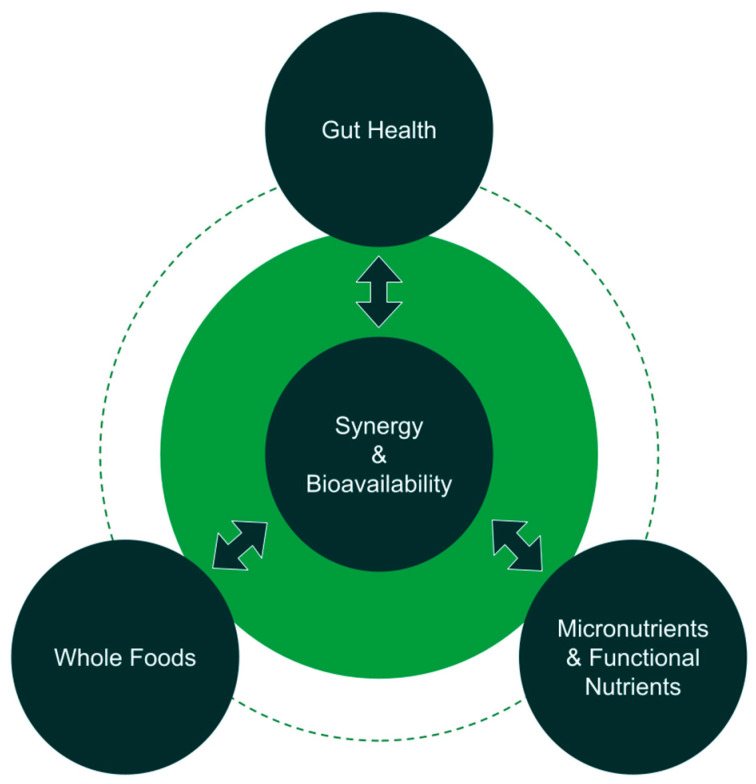
Schematic representation of Foundational Nutrition. Three tenets or pillars of Foundational Nutrition are whole food consumption, micronutrients and other functional nutrients (e.g., phytonutrients), and gut health as key to overall human health. Synergy and bioavailability are themes that connect these three tenets. Specifically, synergy in how nutrients work together, synergy in how these nutrients improve gut health and the function of the digestive system to subsequently deliver nutrients systemically, and how nature provides whole foods (e.g., fruits, vegetables, nuts, etc.) which contain a complex matrix of nutrients that work together synergistically as opposed to being consumed in isolation. Bioavailability also links these pillars in the way bioavailable forms of nutrients are found in whole sources, bioavailable forms of supplemental micronutrients are superior to improving health, and optimal gut health by proxy can increase the bioavailability of nutrient through the microbiome and health of organs in the digestive system.

Therefore, we present Foundational Nutrition as the lens by which we begin to address this complex system with a comprehensive, simplistic, yet synergistic model (Figure 1). This three-pronged Foundational Nutrition approach extends beyond macronutrients, requiring consideration of the following:

### 2.1. Leveraging Nutrient Synergy and Bioavailability

Nature produces foods composed of numerous combinations of macronutrients, micronutrients, phytonutrients, prebiotics, probiotics, and other bioactive compounds. These nutrients do not exist in isolation, but in a matrix of constituents with an array of enzymatic and metabolic targets. Human beings are complex organisms and the reductionistic and mechanistic method by which we are currently assessing nutrients attempts to isolate these systems as separate units and address them independently—separating the part from the whole. Therefore, taking a complex plant extract or food with multiple active constituents and isolating them down to one mechanistic target fails to capture the multi-targeting effects of these nutrients. Instead, the approach to scientific research through the lens of Foundational Nutrition must consider the synergistic effect of multiple biophysiological reactions occurring from these nutrients in a physiological context [18]. When combined, these nutrients have additive and amplified effects that may not necessarily be attributed to one particular constituent or molecular target, but instead is an effect resulting from a concerted effort of multiple different constituents and their affinity for multiple different physiologic targets. For example, the combination of vitamin C and E has been shown to preferentially protect cells from oxidative damage to a greater extent than each nutrient alone [19]. Furthermore, zinc status has been shown to influence vitamin A transport, metabolism, and utilization in the body [20]. We see this effect in the most simplistic form with the inverse relationship between fruit and vegetable intake with chronic disease, and now we must consider the countless other nutrients and their collective benefits through this new lens [21]. The concept of nutritional synergy extends even beyond the interaction of the nutrients themselves. The phenomenon of “nutrient microbial synergy” refers to the mutually beneficial relationship between nutrients and microorganisms in various ecosystems, including the human body [22]. It describes the synergistic interactions where nutrients provided by the diet support the growth and activity of beneficial microorganisms, while these microorganisms, in turn, contribute to nutrient cycling, availability, and overall ecosystem health [23]. Given the prevalence of nutrient deficiencies we will discuss in this review, an emphasis on consuming bioavailable forms of micronutrients may assist in meeting our nutritional needs [24]. One example is that magnesium consumed in the form of leafy greens has a higher bioavailability than those obtained in grain products [25]. Concerning supplementation, magnesium in certain forms (e.g., magnesium glycinate, citrate, chelated) have greater bioavailability in the human body in comparison to other forms of magnesium (e.g., magnesium oxide) [26,27]. We propose that by leveraging synergy and bioavailability concepts, individuals are more favorably positioned to meet their nutritional needs.

### 2.2. Nutrient Quality, Quantity, and Essential Nutrients

Foundational Nutrition incorporates highly bioavailable nutrients that not only includes the basic 26-essential vitamins and minerals, but also argues for a more inclusive approach which incorporates other conditionally essential nutrients (e.g., phytonutrients, omega-3 fatty acids, CoQ10, creatine, etc.), that most seldom find adequately in their diet [28]. Furthermore, we argue for the shift from nutrient quantity recommendations aimed at preventing deficiencies to recommended quantities which promote an improved healthspan and lifespan based on a body of recent, robust scientific research. This requires a comprehensive review of the literature with an emphasis on reassessing nutrient requirements, bioavailability, and quality that has yet to be appropriately considered as a variable in the nutritional health sciences. 

### 2.3. An Emphasis on Supporting Nutrient Absorption through Gut Health

The gut extends beyond just the anatomical structures, but incorporates an ecosystem that when compromised has a profound impact on the function of many other systems. The gastrointestinal system is our body’s primary medium by which we absorb and assimilate various nutrients that we are incapable of synthesizing endogenously. Generally, the microbiome is largely responsible for the breakdown of nutrients to aid in the absorption process (carbohydrates, proteins, bile acids, etc.) and some nutrients require the microbiome or intrinsic factors in the gut to be readily absorbed (e.g., vitamin k, polyphenols, vitamin b12) [29,30,31]. Additionally, microbial dysbiosis decreases the capacity of the small intestine to utilize and absorb nutrients [32]. Commensal bacteria in the gut appear to regulate the expression of nutrient solute carrier transporters demonstrating a potential relationship between gut microbiota composition and mechanistic nutrient absorption [33]. Moreover, gut dysbiosis has been linked to many gastrointestinal disorders (e.g., irritable bowel syndrome, celiac disease, inflammatory bowel disease) as well as systemic conditions (e.g., respiratory issues, cardiovascular disease, metabolic disease, obesity). For example, some reports indicates that 40% of the world population suffers from some form of functional gastrointestinal (GI) disorders such as irritable bowel syndrome (IBS), and it is estimated that 80% of individuals with IBS suffer from small intestine bacterial overgrowth (SIBO) [34,35]. Patients with SIBO experience malabsorption of fat and fat-soluble vitamin deficiencies (D, E, A, and K) which can lead to other health concerns [36]. In addition, approximately 68% of patients with diarrheal IBS had significant bile acid malabsorption [37,38]. Furthermore, probiotic supplementation has been shown to improve nutrient malabsorption in those with gut dysbiosis [32].

Thus, it is reasonable to suggest that in order for humans to improve both lifespan and healthspan, we must address gut health as a priority given the various functions it serves for each organ system and the body as a whole. Therefore, gut health and function is emerging as a key limiting factor in human healthspan and lifespan. To promote health and vitality across all populations, gut health through nutrition should be the center of our focus as we look to the future of nutrition science. 

Together, these three pillars drive Foundational Nutrition and provide a guideline and new model for scientists, clinicians, and the scientific community as a whole to consider as we approach this century where preventive nutritional approaches are essential in the promotion of health and the delay of chronic illness.

## 3. Challenges to an Exclusively Macronutrient-Focused Approach to Nutrition

The World Health Organization (WHO) estimates that 31% percent of the world’s population over the age of 18 are overweight with 13% being obese [39]. In the US, a staggering 66% of adults are overweight and 33% are obese, with those in the “very obese” category rapidly increasing [40]. Not only is the world population overconsuming calories, but we are compounding the negative health effects of excess calories by consuming diets low in micronutrients and phytonutrients. Using the American Heart Association’s diet quality scoring system which takes into account various aspects of a healthy diet (e.g., intake of fruits, vegetables, whole grains, lean proteins, etc.), 45.6% of US Adults consume a diet which is poor in quality [41,42]. As a solution to our low-quality diets, many researchers and practitioners suggest a healthy whole food based diet such as the Mediterranean diet or other plant-based, nutrient-dense diets [43,44,45]. This proves difficult in practice as it is estimated ~75% of the US population (ages ≥1 year) do not consume the recommended intake of fruit, and greater than 80% do not consume the recommended intake of vegetables [46]. Thus, while few would argue with a “foods first” approach to meeting nutritional needs, clearly other options are needed to practically improve human nutrition given the low prevalence of contemporary fruit, vegetable, and whole grain consumption. Furthermore, ultra-processed foods provide 58% of energy intake and 89% of added sugars in the American diet [47]. A recent meta-analysis revealed a significant relationship indicating that diets high in ultra-processed food intake had decreased levels of key nutrients for health (e.g., fiber, protein, potassium, zinc, and magnesium, and vitamins A, C, D, E, B12, and niacin) [48].

These poor dietary choices result in an inadequate intake of essential nutrients such as vitamins, minerals, and other micronutrients. Specifically, data from the National Health and Nutrition Examination Surveys (NHANES) indicate that large portions of the population had total usual intakes (including both food and dietary supplement use) below the estimated average requirement (EAR) for vitamin D (74%), vitamin E (67%), calcium (39%), magnesium (46%), and others [49]. Not only are we consuming unhealthier food as a population, but the food we are consuming is less nutritionally dense than in previous generations [50,51,52,53,54]. A review by Davis and colleagues indicated that the content of six essential nutrients (protein, calcium, phosphorus, iron, riboflavin, and ascorbic acid) in 43 garden crops (primarily vegetables) has declined between 5 and 40% since the 1950s, thus raising another challenge in meeting our Foundational Nutritional needs [50]. Furthermore, a review from Mayer and colleagues in 2022 found nutrient depletion in fruits and vegetables from 1941 to 2019 to be as high as 49 and 50% for iron and copper, respectively, in the UK [52]. In certain areas of the world, nutrient depletion may also be compounded by challenges to the climatic environment [15,55].

The reasons for these declines are multifactorial and regardless of the precise reason, nutrient-depleted diets lead to a range of health problems including diminished immune function, impaired cognitive development and function, anemia, osteoporosis, and even an increased risk of chronic diseases such as cardiovascular disease and certain cancers [56,57,58,59]. Obesity, as a specific example, is characterized by a chronic inflammatory state that is partially brought on by excessive malnourishment [60]. Physiological manifestations of the disease consist of cardiovascular pathologies (e.g., hypertension, atherosclerosis, etc.) and metabolic abnormalities (e.g., dyslipidemia, diabetes, etc.) [61]. Based on the physiological manifestations, it is clear how diet plays a crucial role in obesity. While there is a clear role of excessive caloric consumption that leads to the adipose tissue accumulation, the question of whether malnourishment is a component to obesity becomes compelling to answer. Kobylińska and colleagues outline obesity as a paradox; it can be defined by excessive energy consumption coupled by nutrient deficiencies [62]. This partially occurs because of the consumption of high-calorie foods with low nutrient content [63]. This results in a reported vitamin D deficiency in 80–90% of obese individuals [64,65], with other nutrient deficiencies such as biotin, thiamine, ascorbic acid, cobalamin, folic acid, chromium, selenium, and zinc among others [62,66,67,68,69]. While some argue whether these nutrient deficiencies exacerbate obesity or are a result of obesity, it is undeniable that nutrient deficiencies are occurring. This becomes even more important considering these deficiencies can contribute to the clinical manifestations and secondary disease states of obesity. It is clear that a new approach to attaining a health is needed as we face many challenges from a nutritional perspective.

## 4. History of Nutrient Recommendations and Further Needs

The recommended dietary allowances (RDAs) were first developed in the United States during World War II, stemming in response to the need for a standardized set of nutrient recommendations that could be used to guide public health policies and individual dietary choices [70,71,72]. The first set of RDAs was published in 1941 and included recommendations for nine essential nutrients: protein, calcium, phosphorus, iron, thiamin, riboflavin, vitamins A and D, niacin, and ascorbic acid (vitamin C) [73]. While formulated in the US, these recommendations were soon adopted in various capacities by other nations such as Canada and England and essentially served as the foundation for nutrition policies and recommendations worldwide [73,74]. Over the ensuing four decades, periodic updates to the RDA expanded recommendations to incorporate a wider range of nutrients, including vitamins, minerals, and macronutrients such as carbohydrates, fats, and protein [75]. However, at its inception, the RDA system was designed to provide guidelines for healthy individuals and to prevent nutrient deficiencies and malnutrition, rather than to prevent specific diseases or even optimize health. As such, criticisms arose regarding the limitations of the RDA system, particularly in relation to the lack of consideration given to individual variation in nutrient requirements, and the potential for overconsumption of nutrients at the upper end of the RDA range. 

These critiques led to the introduction of the dietary reference intakes (DRIs) system in 1997 by the Institute of Medicine with the publication of a report establishing a new set of nutrient reference values replacing the RDAs, providing a more comprehensive and nuanced set of nutrient recommendations [25]. One of the key advantages of the DRI system is that it considers the varying nutrient needs of different population groups, including age, sex, and life stage in providing nutrient guidelines which were absent from the RDA system. The DRI system expanded in subsequent years with the publication of additional reports on other nutrients, including vitamin D, vitamin A, selenium, and others [25,76,77,78,79,80,81,82,83,84,85]. The DRI system provides four different types of nutrient reference values, including the RDA, adequate intake (AI), tolerable upper intake level (UL), and estimated average requirement (EAR) explained in more detail elsewhere [74]. 

While the RDA and DRI have been influential in promoting global nutritional health, they are not without limitations. The RDA is determined by defining an intake level that is a “risk for inadequacy” equating to 50% of the population (the EAR). Subsequently, variance is estimated with a generalized variation coefficient of 10%. Finally, two standard deviations at 20% are added to the EAR or the 97.5th percentile of requirements determined by the Monte Carlo simulation [86]. Currently, the RDA/EAR model does not have the capability to predict if a person is biologically deficient in a nutrient, rather, only if they are inadequate from the “notion of adequacy” [87]. As there are a plethora of factors that can impact the unique nutritional needs of an individual (e.g., age, sex, health conditions, genetic polymorphisms, socioeconomic factors, stress, geographic location, etc.), it is impossible to capture all the variables to have an accurate and quality input to yield a proficient statistical RDA model. Revisiting the RDA/EAR using biological data can provide a better model that utilizes biological relevance to determine inadequacy, instead of using mathematical relevance. 

The intent of these guidelines has always been to prevent diseases of nutrient deficiency and never as a proactive approach to chronic degenerative disease prevention let alone optimization. In fact, there have been efforts to try to apply the dietary reference intakes to chronic disease prevention [88] with fluoride, dietary fiber, sodium, potassium, and calcium as the only nutrients with chronic disease endpoints [89]. Outside of these five nutrient disease claims, according to the expert panel, other nutrients do not have substantial evidence for the prevention of chronic disease [90]. As mentioned earlier in this review, chronic diseases such as diabetes, cancer, Alzheimer’s, and cardiovascular disease are largely preventable conditions that arise as a product of poor nutrition [2,3]. 

Periodically, the Food and Nutrition Board (FNB) reevaluates the DRIs and considers adding more nutrients to the DRIs acknowledging there may be other nutritional substances other than the main nutrients for which guidelines should be established [91]. However, the last revision to the DRIs was in 2011 to update recommendations for vitamin D and calcium while no other substantial changes have been made since the initial reports from 1997 to 2004 [25,77,78,79,80,84,85]. A sizable body of new research has emerged in the past decade, and the scientific literature largely suggests that new nutrients and dietary components should be considered as a part of the DRIs, as the evidence suggests these nutrients are necessary for human health [92,93,94]. Omega-3 fatty acids and bioactives such as phytonutrients have been specifically highlighted as nutrients which the DRIs excludes while they exert numerous health benefits and ability to reduce risk of various diseases [92,93,94,95,96]. The DRI system also does not account for nutrient bioavailability which can vary greatly depending on the nutrient and nutrient source (e.g., animal, vegetable, grain, supplement) consumed [25,26,28,97,98]. While the current DRI system accomplishes many basic goals, given the rise of chronic diseases attributable to inadequate dietary practices, it would be prudent for health professionals and policy makers to expand the list of nutrients which hold recommendations for daily consumption to provide a sound nutritional foundation for well-being and healthspan. In the next section, we propose a more inclusive perspective on nutrients which are essential to human health and longevity.

## 5. Nutrients and Foundational Nutrition

Essential nutrients are traditionally defined as an organic compound that serves a crucial physiological function in the human body and cannot be synthesized endogenously in humans (e.g., ascorbic acid); whereas non-essential nutrients may support a structure or function in the body but may be endogenously produced by other nutrient precursors in the body and are dependent on rate of conversion or metabolic processes internally. While these definitions provide a framework to understand the detrimental effects of nutrient underconsumption (e.g., malnutrition), it appears to fall short in establishing conditions for optimal nutrition over time to promote both healthspan and lifespan. For instance, the body of nutritional scientific literature provides numerous examples of non-vitamin or mineral nutrients which when consumed in sufficient amounts and in combination with other nutrients have profound effects on human health and longevity including various phytonutrients, omega-3 fatty acids, microbiome metabolites, and others. Moreover, the strength of the evidence is such that these and other functional nutrients (e.g., co-enzyme Q10, alpha-lipoic acid, etc.) should be included in our discussion of outlining the constituents of foundational nutrients (Table 1).

Phytonutrients are a broad array of naturally occurring compounds found in plants, including those found in adaptogens, functional mushrooms, and a variety of whole foods, which exert numerous beneficial biological effects when consumed through the diet [99]. Thousands of phytochemicals have been identified in plant matter, which have been generally categorized into several groups including phenolics (polyphenols), alkaloids, nitrogen-containing compounds, organosulfur compounds, phytosterols, and carotenoids [100]. The primary classes of polyphenols consist of phenolic acids, flavonoids, stilbenes, and ligands [101,102]. While there are extensive reviews of the positive effects of polyphenol consumption [103,104,105,106], in short, these bioactive compounds have been demonstrated to serve as intra- and extracellular antioxidants, stimulate microbial diversity, beneficially modulate the immune system, provide neuro-protective effects, and target multiple mechanistic pathways to enhance human health [107,108,109,110]. More specifically, polyphenol interventions have been shown to have immunomodulatory properties by decreasing pro-inflammatory cytokine production (e.g., IL-6, TNF-ɑ) in vitro and in human models [107,111,112,113]. Inflammatory cytokines play a crucial role in the immune response and can contribute to the development and progression of various diseases [114,115,116]. A meta-analysis including 30 RCTs and 5166 participants revealed significantly beneficial effects of polyphenol administration on decreasing illness intensity and the sum of symptom ratings in those experiencing viral acute respiratory tract infections [117]. A citrus bioflavonoid, hesperidin, has been shown to provide immune and cardiovascular benefits by increasing flow-mediated dilation, reducing blood pressure, and attenuating c-reactive protein (CRP) compared to a placebo [118,119,120]. Regarding obesity, polyphenols such as epigallocatechin gallate (EGCG) have been shown to positively influence reductions in adiposity likely by elevating energy expenditure and lipolysis [121,122,123]. Data also suggest polyphenols may be an effective means to suppress sensations of hunger and promote feelings of fullness and satiety which may contribute to observed reductions in fat mass [124].

Coenzyme Q10, also known as CoQ10, is a naturally occurring compound found in the mitochondria and serves an important role as an energy transfer molecule in the production of adenosine triphosphate (ATP) [125]. It also functions as a potent antioxidant, providing protection to cellular membranes from damage caused by free radicals. Given these antioxidant properties, it appears CoQ10 may provide a protective effect against cardiovascular disease by reducing the oxidative potential of low-density lipoprotein (LDL) particles which contribute to atherosclerosis [126,127]. Furthermore, a meta-analysis of 17 trials found that CoQ10 supplementation significantly reduced systolic blood pressure in patients with other metabolic diseases [128]. Moreover, animal models indicate that CoQ10 may help to improve brain health by improving cerebral blood flow, enhancing cognition, and attenuating impairments from neurological disorders. The antioxidant properties of CoQ10 may also support skin health by reducing the visible signs of aging and skin elasticity [129]. Regarding chronic inflammation, a meta-analysis found that short- and long-term CoQ10 administration taken in doses ranging from 60 to 500 mg/day significantly decreased production of inflammatory cytokines (e.g., C-reactive protein, tumor necrosis factor alpha, interleukin 6) which are commonly linked to various pathologies [130]. Overall, CoQ10 is a powerful nutrient that may offer numerous health benefits, particularly for heart health, energy production, and geroprotection.

Omega-3 fatty acids are a group of polyunsaturated fatty acids consisting of alpha-linolenic acid (ALA), eicosapentaenoic acid (EPA), and docosahexaenoic acid (DHA) [131,132]. ALA is considered an essential fat since the body does not have the necessary enzymes for endogenous synthesis; however, recent data suggest that the US population generally consumes the recommended amount through their normal diet, commonly through seeds and nuts [133]. While a dietary reference intake has been established for ALA of 1.6 g and 1.1 g for men and women, respectively, the same cannot be said for EPA and DHA. Most health organizations suggest that humans consume approximately 250 to 500 mg of EPA and DHA combined. Given that ALA conversion into EPA and DHA is limited to approximately 15% [134], this would suggest that the average human would have to consume approximately two to three times the amount of ALA to meet the minimal EPA and DHA recommendations, not considering the vast amount of literature suggesting benefits of at least 1 g of EPA and DHA daily [135]. Consequently, the NIH recommends consuming EPA and DHA from whole food sources and/or dietary supplements to achieve adequate amounts in the diet [136]. Yet, a recent cross-sectional study indicated that 95% of US children and 68% of adults had serum omega-3 status below what is recommended by the Dietary Guidelines for America indicating underconsumption of omega-3 foods [137]. Omega-3 fatty acids have been shown to inhibit very low-density lipoprotein and triglyceride synthesis in the liver, decrease platelet-derived growth factor production and messenger RNA synthesis, and free radical production in neutrophils which contribute to improved endothelial health and attenuation of CVD [138,139,140]. Specifically, a large-scale clinical trial (*n* = 8179) found that consuming 4 g/day of a purified EPA omega-3 supplement for ~5 yrs significantly reduced the risk of experiencing ischemic events and cardiovascular death in individuals ≥ 45 yrs with CVD [141]. Further, a recent meta-analysis of 13 clinical trials administering between 0.376 and 4 g of omega-3 supplements per day indicated that omega-3 supplementation significantly reduced the risk of myocardial infarction, coronary heart disease (CHD), CHD related death, CVD related death, and total incidence of CVD in a dose-dependent manner [142]. Regarding cognition, another meta-analysis found that omega-3 administration significantly improved various domains of cognition (e.g., immediate recall, attention, processing speed) in adults with mild age-related cognitive impairment compared to a placebo [143]. Given the other reported benefits of omega-3 consumption on reductions in major adverse cardiovascular events [144], cancer development [145], Alzheimer’s and cognitive decline [146], depression [147], rheumatoid arthritis [148], and many other conditions [149], it is clear that these biological compounds are powerful agents in promoting human wellness across the lifespan and must be considered a part of one’s Foundational Nutrition.

While not traditionally defined as nutrients, substantial evidence has emerged over the past two decades indicating the profound health effects of probiotics and probiotic-containing foods for promoting gut microbiome community diversity [150]. Recent data suggest that these multifaceted bacteria and their metabolites (short chain fatty acids, tryptophan metabolites such as indole, aryl hydrocarbon receptor ligands, polyamines, etc.) have the potential to modulate the immune system, enhance nutrient availability, and influence other health domains (e.g., neurological, musculoskeletal, reproductive systems) [32,151]. Taken together, several prebiotic, probiotics, and their subsequent metabolites should be deemed “nutrabiotics” given their pleiotropic health benefits and the lost benefit on health and longevity in their absence [152]. Pre- and probiotics might also play a beneficial role in managing obesity. While obesity is not the only challenge to human health, it commonly manifests as a result of poor nutrition and is generally seen as a gateway to many other diseased states including type 2 diabetes, cardiovascular disease, non-alcoholic fatty liver disease, and hypertension [39,153,154,155]. Some evidence exists that prebiotics can promote feelings of satiety and promote weight control [156,157]. Moreover, the usage of prebiotics and probiotics has been shown to have success in clinical studies involving obese individuals [158,159,160,161]. A study by Niccolucci and colleagues in 2017 demonstrated that oligofructose-enriched inulin was able to reduce fat mass and body weight, alter fecal bile acid content, and alter the gut microbiome composition in overweight and obese children [160]. Specifically, these physiological outcomes appear to occur through modulation of *Bifidobacterium* spp., resulting in decreases in *Bacteroides vulgatus* [160]. While this is just one example, meta-analyses show that composition changes do occur that lead to meaningful physiological changes including changes in the inflammatory status of obese individuals. Beyond just obesity, it is generally understood that pre-/probiotics do contribute to an overall anti-inflammatory status as well as exert immunomodulatory effects [162,163]. The StatPearls page “Dietary Approaches To Obesity Treatment” has no mention of polyphenols, pre-/probiotics, or phospholipids and briefly mentions phytochemicals once with no follow-up or discussion [164]. Therefore, the purpose of Foundational Nutrition as a modern lens to view nutrition and nutritional science is to bring these dietary components to the foreground so they are discussed directly.

Mental health and high levels of stress are being reported at increasing rates globally [165,166,167]. While some nutrients within many current nutritional guidelines have been positively associated with mental health and stress, such as vitamin B9, B6, B12, and folate [4,8,168], other compounds that are not currently under nutritional guidelines that support mental health exist, such as phospholipids and specific bioactives found in botanicals. In vitro experimentation demonstrates that phospholipids can be neuroprotective and even regulate inflammatory processes through specific cell types like astrocytes [169]. With time, clinical studies are also emerging showing some clinical success between specific phospholipids and the treatment of mental disorders. Komori in 2015 saw that 100 mg of phosphatidylserine, 119 mg of docosahexaenoic acid, and 70 mg of eicosapentaenoic acid taken three times daily for 12 weeks was successful at managing depression in older individuals by regulating cortisol homeostasis [170]. Furthermore, bioactive phytonutrients found in *Rhodiola rosea* were shown to reduce symptoms of mild to moderate depression, mild anxiety, and provide a mood enhancing effect in a recent meta-analysis [171].

Although it is outside the scope of this article to review every pertinent nutrient to Foundational Nutrition, there are many other nutraceuticals which have garnered attention for the growing evidence regarding benefits to human health, longevity, and resiliency to stress. While not an exhaustive list, some compounds for nutrient consideration are: dietary nitrates, creatine, alpha-lipoic acid, adaptogens (e.g., Ashwagandha, Eleuthero, mushroom compounds), beta-alanine, quercetin, and citrulline [172,173,174,175,176,177,178,179,180]. Nevertheless, in re-examining the nutritional needs of an ever-changing population with unique health challenges, we propose that health professionals expand their definition of “essential nutrients” to help various populations attain the goal of comprehensive nutrition that aligns with the evolution of the scientific literature since the first RDA in 1941.

## 6. Importance of Gut Health

Nutrients, regardless of whether they come from diet or supplemental sources, go through various stages of digestion and absorption via the gastrointestinal system. It is a complex, multi-organ system with a nuanced communication system between human cells and microbial organisms. Loosely, the digestive system begins in the mouth and proceeds downward to the stomach and subsequently to the small and large intestines where the majority of the resident microorganisms aid in the digestive and metabolic processes. After the last stages of absorption, unabsorbed nutrients and waste are excreted from the body. During digestion, the amount of nutrient absorption can be modified due to biological processes in site-specific mechanisms.

The gastrointestinal tract is necessary for nutrient absorption, assimilation, and utilization, but it is not sufficient when assessing the necessities for Foundational Nutrition. Chiefly, the nutrient slurry that passes through the intestines encounters the gut microbiome which includes numerous microbial organisms across several domains of life. Bacteria, archaea, viruses, fungi, and other eukaryotic organisms play critical roles in shaping the microbial ecosystem. [181,182,183,184,185]. This rich, biodiverse microbiome which spans several domains of life leads to an array of metabolic processes that cannot occur due to limitations in the human genome. In many cases, this is crucial for human health as some microbial metabolic byproducts are key nutrients for humans. Half of a person’s necessary vitamin K comes directly from gut microbial metabolism [31] and specific intestinal epithelial transporters aid in the absorption of microbial derived nutrients [186] that would otherwise be excreted (e.g., various phytonutrients).

Several factors influence the gut microbiome’s composition, but nutrient availability, and conversely nutrient deficiency, are key factors [187]. Since nutrient availability shapes the microbial ecosystem, having a foundational and robust selection of nutrients is vital to maintain the array of metabolic processes that nourish the host. One study showed that malnourishment directly shifted microbial communities and resulted in sequential metabolic deficiencies which were detected in the host’s serum [188]. If nutrient deficiencies result in microbial-induced serum deficiencies, then it is possible that optimal nutritional states confer microbial-induced optimum serum concentrations. This, however, remains to be fully explored. While diet is generally understood to shift microbial composition, no study directly explores this phenomenon to date.

Beyond the gut microbiome, the cellular processes and organ systems can influence nutrient availability prior to nutrient delivery at the target site. Some nutrients can influence enzyme kinetics and even enzyme numbers. Optimal levels of a nutrient can change the substrate affinity of transport enzymes [189] or even upregulate enzyme expression [190]. As these cells work in concert in the organ system, optimal nutrition can amplify vital organ function. In the case of the liver, vitamins and minerals are critical in maintaining its histological integrity and catalytic function [191]. Damage to these organs can inhibit enzymatic activation of vital nutrients or result in lower nutrient stores [192]. Together, changes in enzyme number or kinetics and tissue integrity can influence nutrient availability at the target site. Experimental data show that these impairments, even at the gut level, can potentiate this effect systemically [193].

Microbiota disturbances are commonly noted in many disease states, but particularly in obesity. The obese gut microbiome is described as being low in taxonomic diversity [194,195] and in abnormal metabolic function [196,197]. Although, these findings are not always consistent from study to study [198], a recent meta-analysis by Pinart and colleagues examined 32 studies as well as other meta-analyses to describe the obese gut microbiome composition. Six of the studies indicated that there was a higher abundance of Firmicutes (F) with fewer abundances of Bacteroidetes (B), thus a characteristically high F:B ratio is commonly observed. At the genus level, there is a lower reported abundance of *Bifidobacterium* and *Eggerthella* in the obese gut microbiome; a higher abundance for *Acidaminococcus*, *Anaerococcus*, *Catenibacterium*, *Dialister*, *Dorea*, *Escherichia-Shigella*, *Eubacterium*, *Fusobacterium*, *Megasphera*, *Prevotella*, *Roseburia*, *Streptococcus*, and *Sutterella* were reported for the obese gut microbiome [199]. It is generally understood that these microbial disturbances in the gut microbiome contribute to the pathophysiology of obesity [147,200,201]. However, beyond just obesity and as previously mentioned, the gut microbiome plays a complex role in maintaining human physiology, homeostasis, and engages as a first responder to human diet and nutrition resulting in significant changes in nutrient absorption and human metabolism.

**Figure 2:**
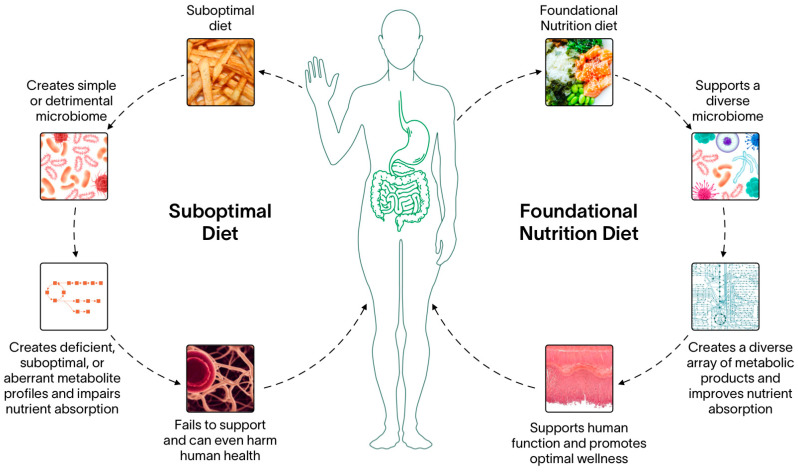
Foundational Nutrition implications on gut microbiome health. Human diet is one of the most important variables that dictates the community composition of the gut microbiome. Suboptimal diets that are generally lacking in robust nutrients sources and are highly processed reduces the nutritional inputs the gut microbiome receives. Consequently, the gut microbiome will shift towards a metabolically simplistic composition and reduce global diversity. Reductions in community composition can lead to reductions in metabolic diversity, which has been shown to reduce the amount of some nutrients in human serum which can lead to systemic abnormalities and even become precursors to disease states. Conversely, supporting the gut microbiome with a diverse input of nutrients supports a wide array of microbiota, including rare taxa whose metabolism is critical in maintaining human homeostasis. A diverse microbiome community composition leads to a diverse metabolite composition which is bioavailable for the host to utilize. This keeps the body’s physiology functional and promotes global well-being.

Digestion is a process with several potential bottlenecks. However, it could also serve as a mechanism to increase nutrient bioavailability. By increasing nutrient density in food and revisiting nutrient values that promote optimal biological processes beyond just homeostatic functions, the nutrient pool available to perform cellular work increases. These revisions could theoretically amplify the essential metabolic byproducts the gut microbiome provides to their host. Biochemically, optimal nutrition could increase nutrient affinity, leading to increased cellular and serum nutrient levels that can support organ function throughout the body. Synergistically, these work in concert to not only maintain human biology but promote vigor and vitality (Figure 2).

## 7. Merely the Beginning

Considering the health challenges facing our world population, the need is clear for continually improving recommendations and strategies to promote human health. The purpose of this review was to show that this global problem, and the clear lack of any immediate resolution, is indicative that the current lens with which the nutritional science community approaches optimal human nutrition is lacking. The concept of Foundational Nutrition prioritizes key pillars which have a profound impact on every health system in the human body. An emphasis on consuming highly bioavailable nutrients which exert synergistic effects elicits a greater impact on overall health than the sum of their individual or less-potent nutrient effects. Furthermore, consuming a diet rich in micronutrients and other phytonutrients not traditionally included in nutritional guidelines plays a key role in maintaining a healthy immune system, preventing chronic diseases, promoting optimal cognitive function, and countless other health benefits. Finally, in view of the myriad of health problems and diseased states which have been linked to poor gut health, Foundational Nutrition designates gut health as a focal point to improve systemic health and nutritional status. It is time to consider that the current nutrient crisis is not a problem of just a few nutrient deficiencies; rather, it is a concert of multiple factors leading to a global reduction in the human quality of life.

With this review, we hope to spark a new conversation around how society views human nutrition and how the scientific community approaches research on the topic. By opening up the space to have scientific discourse on how nutrients are viewed in the biological system, we can begin to elucidate the importance of a diverse diet in terms of nutrient composition. By reevaluating how society views nutrition, it is possible to pave the way to a solution of a problem that is only growing within the current frame of thinking. Only by accepting that modern food and the guidelines by which they are followed are not enough, can we, as a society, begin to tackle this global crisis.

## Figures and Tables

**Table 1:** Comparison of nutrients included in the recommended daily allowances (RDAs) and dietary reference intakes (DRIs) vs. those proposed for consideration in the framework of Foundational Nutrition.

Nutrients in Current RDA and DRI		Nutrients for Foundational Nutrition	
**Macromolecules**			
Lipids (Total fat, Cholesterol, etc.) Carbohydrates (Total sugars, Fiber, etc.) Proteins		Lipids (Total fat, Cholesterol, etc.) Carbohydrates (Total sugars, Fiber, etc.) Proteins	
**Vitamins and Minerals**			
Vitamin E Vitamin C Vitamin B12 etc.	Zinc Sodium Copper Iron etc.	Vitamin E Vitamin C Vitamin B12 etc.	Zinc Sodium Copper Iron etc.
**Phytonutrients**			
		Phenolic acids Flavonoids Lignans Stilbenes	
**Prebiotics and Probiotics**			
		Fermentable fibers (fructans, beta-glucans, pectins, etc.) Lactic acid Bacteria (*Lactobacillus* spp., *Lactococcus* spp., *Bifidobacterium* spp, etc.)	
**Functional Nutrients**			
		Coenzyme Q10 Alpha Lipoic Acid Phospholipids etc.	

## Data Availability

No new data were created or analyzed in this study. Data sharing is not applicable to this article.
